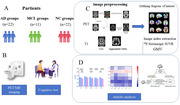# Amyloid and tau PET as well as functional connectivity mediate the association between APOEε4 and neurodegeneration

**DOI:** 10.1002/alz.087280

**Published:** 2025-01-09

**Authors:** Bing Zhang, Xinru Xu

**Affiliations:** ^1^ Department of Radiology, Nanjing Drum Tower Hospital, Affiliated Hospital of Medical School, Nanjing University, Nanjing China; ^2^ Department of Radiology, Drum Tower Hospital, Clinical Colledge of Nanjing Medicial University, Nanjing 210008, china, Nanjing China

## Abstract

**Background:**

It remains unclear to what extent APOEε4 relates to amyloid beta (Aβ) and tau deposition, functional connectivity and neurodegeneration in Alzheimer's disease spectrum.

**Method:**

In 345 participants including subjective cognitive impairment and mild cognitive impairment and Alzheimer disease, We investigate the association of APOEε 4 with Aβ and Tau, functional connectivity and neurodegeneration (cortical thickness and gray matter volume). Using mediation analyses, we tested the indirect effects of APOEε4 on neurodegeneration via Tau deposition (^18^F‐AV451 positron emission tomography) and interregional functional connectivity (functional MRI) in Braak I‐II and Braak III‐VI.

**Result:**

We observed that increased APOEε4 dose was associated with poorer neurodegeneration in AD typical regional, with the strongest effects observed for medial temporal lobe gray matter atrophy. These relationships were mediated mainly via tau pathology, particularly of the medial parietal lobe, and to a lesser extent serially mediated via Aβ and functional activities in AD signature regions.

**Conclusion:**

In summary, our finding highlight a pathway in which APOEε4 affects neurodegeneration via increased tau pathology and reduced functional connectivity in critical lobe. Examining APOE ε4‐related brain changes through the use of neuroimaging biomarkers in the Alzheimer's disease spectrum provides a unique opportunity to advance our knowledge of the underlying pathophysiology of AD, particularly in the stage of subjective cognitive decline (SCD).